# Long-term follow-up of keystone perforator island flap in reconstructed myelomeningocele defects^✰^

**DOI:** 10.1016/j.jpra.2023.09.008

**Published:** 2023-10-02

**Authors:** Tetyana Kelly, James Leong

**Affiliations:** a Plastic and Reconstructive Surgery Department, Monash Children's Hospital, Melbourne, Australia; b Department of Surgery (School of Clinical Sciences at Monash Health), Monash University, Melbourne, Australia

**Keywords:** Myelomeningocele, Spina bifida, Keystone flap

## Abstract

Myelomeningocele (a type of spina bifida) is the most common congenital condition that causes lifelong physical disability and requires multi-system surgical procedures. Therefore, it is paramount to reconstruct them using a stable and robust method that heals with minimal wound issues and produces maximum durability.

We published a case series on reconstruction of myelomeningocele defects using keystone perforator island flap in the *Annals of Plastic Surgery* in 2016.^1^ We aim to report the results of long-term follow-up of 14 years on our case series, where we assessed their scars using POSAS 3.0. We also assessed their quality of life using the QUALAS tool for teenagers.

While there are multiple reconstructive options for the closure of myelomeningocele defects, we believe that the keystone perforator island flap technique is reliable and safe as it utilizes the en bloc movement of a large flap of well-vascularized skin, cutis, and muscular fascia to close large defects in the lumbar-sacral regions in newborns, resulting in stable scars.^2–7^

## Background

Spina bifida is a congenital condition defined as a failure of neural tube formation during early embryonic development that results in several types of spina bifida, with the most common type being myelomeningocele, in which both meninges and spinal cord herniate through the vertebral defect.^1^ The severity of myelomeningocele varies from minor to major depending on the associated medical conditions, which may include paralysis, cognitive impairment, and bladder and bowel dysfunction. Most open neural tube defects are closed within 48 h of birth, which involves a combined effort from neurosurgeons, who perform the pseudo-dural sac closure over the placode, and plastic surgeons, who provide overlying of soft tissue cover. Many reconstructive options exist for the soft tissue repair of this congenital defect, including split skin grafts and multiple types of local and regional flaps, which include rotation, advancement, bipedicled, bilobed, transposed muscle flaps (latissimus dorsi, gluteus, and trapezius), and perforator flaps (superior gluteal artery perforator and dorsal intercostal artery perforator).^2^, ^3^, ^4^, ^5^, ^6^, ^7^ The complications associated with the above reconstructive options include wound dehiscence, wound infection, cerebro-spinal fluid (CSF) leak, CSF infections, and painful, insensate, and unstable scars.^8^^,^^9^ More recently, intrauterine fetoscopic closure of myelomeningocele has been used in some centers and has shown promising results in postnatal psychomotor development and walking.^8^^,^^10^^,^^11^ However, this technique is highly complex and not widely available.

Keystone perforator island flaps (KDPIF) were first described by Felix Behan^12^^,^^13^ for soft tissue reconstruction. “The KDPIF is a curvilinear shaped trapezoidal design flap,” which is essentially two V–Y flaps placed end to side.^12^ Smaller defects can be closed using a single flap, whereas larger defects may require a double keystone flap. The keystone flap incorporates multiple fasciocutaneous and muscular perforators making it very robust and reliable. Its application in the reconstruction of myelomeningocele is described in several international case series.^14^, ^15^, ^16^, ^17^, ^18^, ^19^

We have previously described in our series that using keystone flaps is the preferred surgical technique for the repair of myelomeningocele at Monash Children's Hospital, Melbourne, Australia.^20^ This paper describes the long-term scar outcomes of the original cases.

## Methods

### Ethics

This study was approved by the local institutional ethics committee of Monash Health (HREC reference number RES-22-0000-154Q-84925).

### Study population

All five patients from the original case series were invited to participate in the long-term follow-up study via phone call or email (Table 1).^21^ The family of patient one did not want to participate; hence, that patient was excluded from the study. For those who agreed to participate, we organized a long-term follow-up via face-to-face appointment in spina bifida multidisciplinary clinic or via Telehealth if the patient had moved interstate or could not physically attend the clinic.

**Table 1:** Patient demographics from the original case series.1

Patient	Gestation(weeks)	Sex	Age (days)	Weight (g)	Level	Defect size (cm^2^)	Type of repair	Length of stay (days)
1	38	M	1	3285	S1-S5	100	Bilateral	14
2	33	M	1	1920	S2-S3	16	Bilateral	22
3	38	M	1	2851	L5-S1	25	Bilateral	16
4	40	F	3	3230	S1-S5	8	Unilateral	18
5	30	M	1	1190	L5-S1	16	3-flaps	84

### Study design

**Figure 1:**
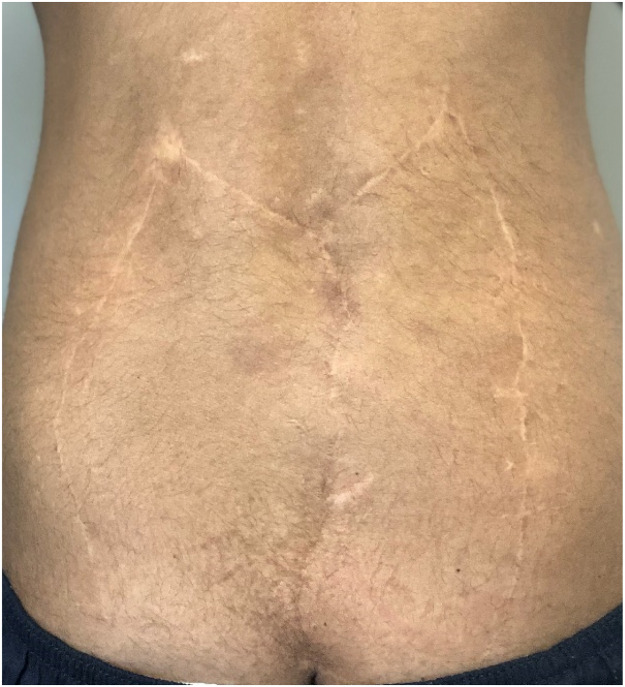
Patient 2 from the original case series.

**Figure 2:**
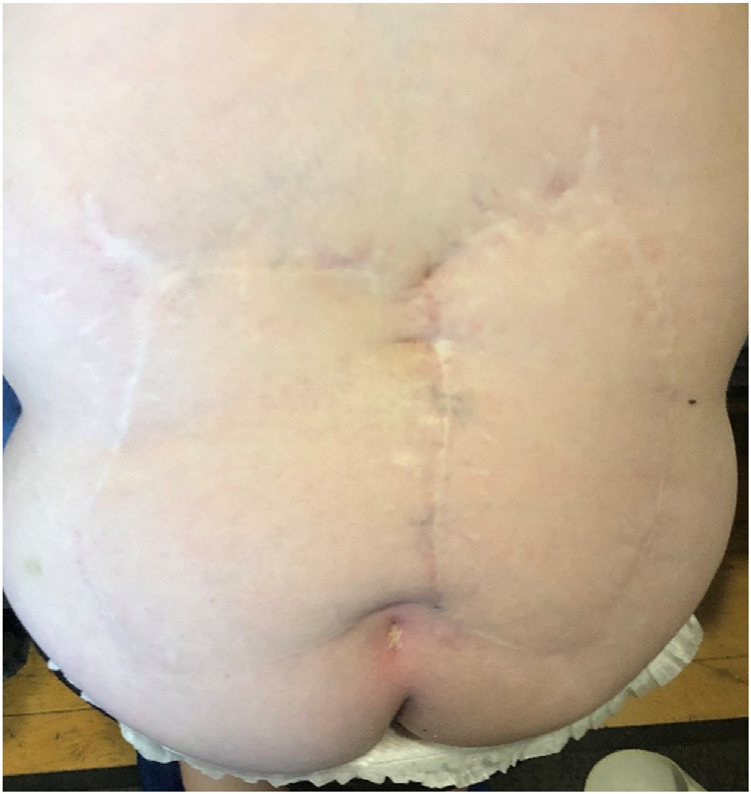
Patient 3 from the original case series.

**Figure 3:**
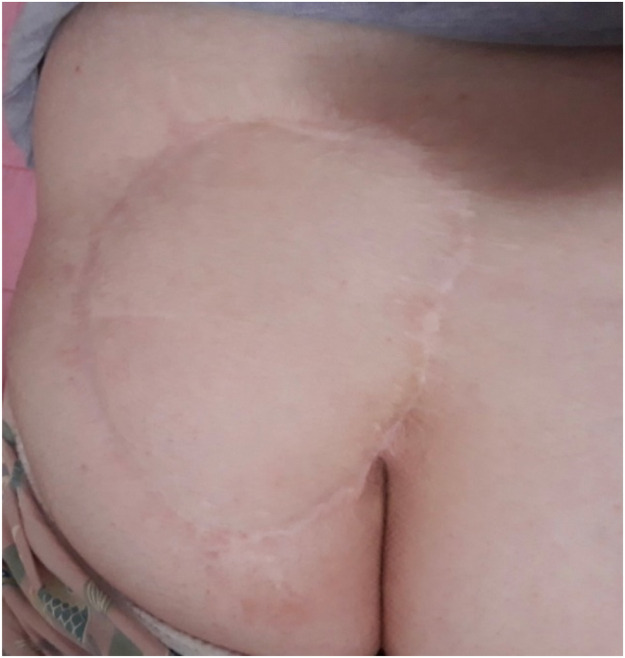
Patient 4 from the original case series.

**Figure 4:**
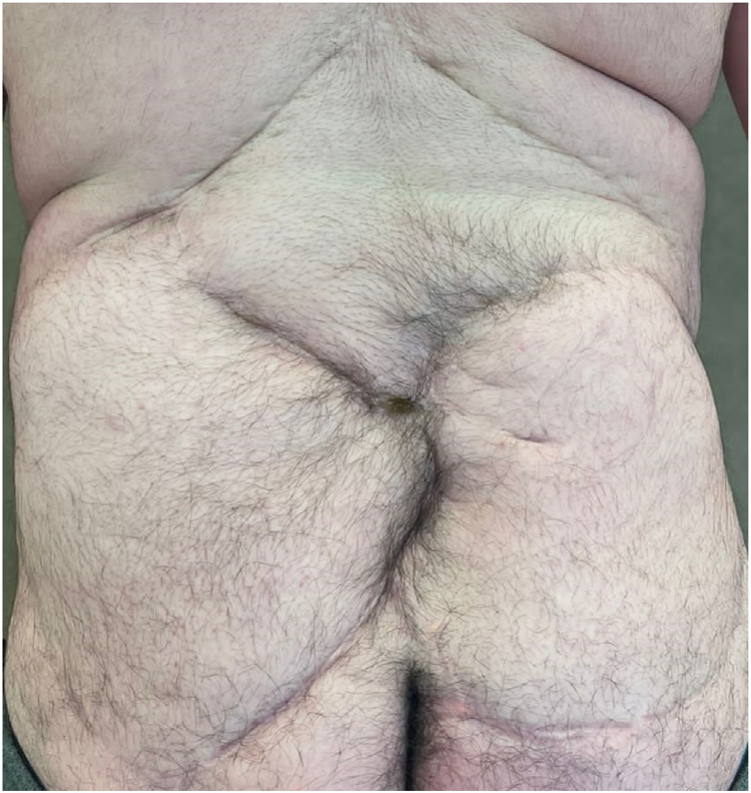
Patient 5 from the original case series.

The study comprised a scar assessment and quality of life questionnaire. After gaining consent, we obtained photographs of patients' backs using an iPhone and Samsung phone camera in the spina bifida clinic or received photographs taken using their parents' camera by email for analysis (Figure 1, Figure 2, Figure 3, Figure 4). All photographs were cropped to a minimum of 3000 × 4000 pixels, and printed in color in size A4 for scar assessment. The questionnaires were distributed during Telehealth or face-to-face appointments in the spina bifida clinic and answered by participants with or without their parent's or guardian's help. The follow-up period was 12–14 years.

### Patient and observer scar assessment scale

We evaluated and measured the quality of the scars of our patients using the validated tool patient and observer scar assessment scale (POSAS) version 3.0. The POSAS measures scar quality from the perspectives of the patient and clinician. We used a modified version without the patient arm considering the age of the participants and the lack of validity in this cohort. The observer arm (Linear Scar version 3.0) of this measurement scale assesses scar vascularity, pigmentation, thickness, relief, pliability, and surface area. It has 10 questions with scores from 1 to 5 points (normal to highly abnormal when compared to normal skin), therefore the smaller the total score the better the scar. All scars were scored based only on photographs to standardize the assessments, especially for those who moved interstate and could not attend the clinic. The assessors could not provide a score on the tactile questions, and hence those questions were not counted in the final score. We asked two independent plastic surgeons and two occupational therapists who specialize in scar management, to assess and score the scars using the recent photographs of four patients.

### QUAlity of Life Assessment in Spina bifida for Teenagers (QUALAS-T)

We used the QUALAS-T survey for the assessment of the adolescent quality of life. This tool has two domains with five questions each assessing aspects of family, independence, and bladder and bowel function affecting teenagers’ lives. Each domain has a maximum score of 100 points if one has the best quality of life.

### Statistical analysis

Descriptive statistics were used to describe the study population.

## Results

### POSAS

The observers were able to answer 7–9 questions from the observer scale when assessing the photographs. We scored the same seven questions (range 7–35, with 7 being the best and 35 being the worst) for all assessors. The maximum score in our modified method was 15 and the breakdown of the scores is presented in Table 2.

**Table 2:** POSAS (patient and observer scar assessment scale) results https://www.posas.nl.

Fig	Plastic surgeon 1	Plastic surgeon 2	Therapist 1	Therapist 2
1	10	12	14	14
2	14	13	14	12
3	15	15	14	10
4	13	15	14	15

None of the patients had any keloid, painful, or itchy scars. The overall quality of the scar showed minimal difference when compared to unaffected skin. Pigmentation was also minimal in most scars. All scars were scored as pale or pink, without the documentation of a purple or red scar. The surgeons were more critical of the visibility of the marks from surgical wound closure compared to therapists.

### QUALAS-T

QUALAS-T has two domains: family & independence and bladder & bowel with a total of 10 questions scoring from 0 to 20 points (normal to highly abnormal). The smaller the total score the better quality of life when compared to adolescents without spina bifida. The breakdown of scores from the questionnaire are presented in Table 3. The scores ranged from 40 to 140. The higher scores reflect the severity of disability of a child with spina bifida.

**Table 3:** QUALAS-T (QUAlity of Life Assessment in Spina bifida for Teenagers)^22^ results.

Patient	Family & independence	Bladder & bowel	Total
1	5	60	15
2	20	65	40
3	25	30	90
4	50	50	140

## Discussion

Lacobucci et al.^23^ while describing the reconstruction of large myelomeningocele using bilateral bipedicled paramedian fasciocutaneous flaps, identified three main vascular territories of the back: parascapular and scapular branches of circumflex scapular artery superiorly, muscular perforators, and cutaneous branches of intercostal arteries in the lumbosacral area. Unlike perforator-based fasciocutaneous flaps, the keystone flap in the back region includes multiple musculocutaneous and fasciocutaneous perforators. The blood supply is from all eight lumbar arteries, posterior intercostal and subcostal arteries directly from the thoracic aorta, thoracodorsal artery via latissimus dorsi, and superior gluteal arteries.^19^ Several types of keystone flaps have been described by Behan. In type 1 flaps, the deep fascia is preserved, which is suitable for small defects. In type 2a flaps, the deep fascia along the outer curvilinear incision is divided allowing further advancement; in type 2b, the deep fascia is divided and the keystone donor defect is skin grafted; type 3 comprises bilateral flaps; and type 4 has flap rotated and raised up to 50% subfascially.^12^^,^^24^

In our case series, we used type 2a for small defects, type 3 for moderate to large defects, and type 4 in a case with a very large defect.

The Monash Health plastic surgery department has pioneered the use of the keystone flap technique to reconstruct myelomeningocele soft tissue defects.^25^ This is the first case series that describes the 12–14 years long-term follow-up of patients with KDPIF reconstruction of myelomeningocele.

Because of its versatility, KDPIF has been used in all age groups. There are several case series describing the application of keystone flaps in children with myelomeningocele defects and a few case reports and case series describing keystone flap technique in children for the reconstruction of giant congenital nevi, vascular malformations, Fournier gangrene defect, and traumatic Morel–Lavallee lesion.^26^^,^^27^ While a vast amount of research has been published on outcomes in adult patients, a case series with long-term follow-up in children have not yet been published.

KDPIF provides stable, well-vascularized, soft tissue reconstruction with short surgical time, and no complex postoperative monitoring when compared to perforator-based fasciocutaneous flaps.^17^^,^^24^^,^^26^ Keystone flap application resulted in reduced morbidity, mortality, and hospital stay.^26^ Moreover, functional and cosmetic results of KDPIF, its versatility, and low complication rates supersede that of any random pattern perforator flap.^28^

Long-term follow-up of children with spina bifida and their transition into adult services have been studied as part of the quality of life assessment.^22^, ^29^, ^30^, ^31^ While previous studies suggested that walking and survival are predicted by the level of lesion, other studies claim that living independently in the community is attributed to the CSF shunt history.^29^, ^30^, ^31^ Overall, median survival for a cohort of 116 patients was 29 years; however, it is better when the defects involved were below L 3 sensory level compared to those above L 3 sensory level (54% vs. 22 %, *p* = 0.001). Three out of four of our patients had a history of CSF shunt insertion shortly after their myelomeningocele repair. The robust predictable healing facilitated by soft tissue coverage has enabled early and safe CSF shunt placement, which is a paramount factor for living independently.

The authors chose QUALAS-T because it is a validated health-related quality-of-life tool for adolescents with spina bifida and it is simple and reproducible, which is crucial when working with children and their families.^32^

We used POSAS because it was found to be superior in performance compared to the other 17 scar scales according to systematic reviews published in 2004 and 2012.^33^ In our study, plastic surgeons and therapists scored patients similarly and the major difference was in the assessment of the marks visible from surgical closure.

## Conclusions

Since its original design, several variations of KDPIF have been described. KDPIF has demonstrated consistency in promoting blood supply and wound healing, which confirms its robustness in the reconstruction of any wound defect.

Unlike, the classic perforator flap, KDPIF surgical technique does not require identifying and dissecting any specific perforators, owing to which it is associated with a reduction in complexity and associated morbidity.^26^

Patients whose myelomeningocele were reconstructed using KDPIF over a decade ago showed robust and stable scars, without pain or itching, and their quality of life was not affected.

In conclusion, our study demonstrates that reconstruction using KDPIF is a reliable and robust surgical technique that can facilitate the closure of myelomeningocele defects, which are 8–100 cm**^2^** in size, in newborns with spina bifida, providing a robust, non-itchy, non-painful, and aesthetically acceptable reconstruction well into adolescence.

## Declaration of Competing Interest

None.
